# The Breaking Strain

**Published:** 1896-05-23

**Authors:** W. H. Allchin

**Affiliations:** Physician to the Westminster Hospital


### Medical Progress and Hospital Clinics.

[*The Editor will be glad to receive offers of co-operation and contributions from members of the profession. All letters should be addressed to* The Editor, at the Office, 428, Strand, London, W.C.]

### THE BREAKING STRAIN.

Oration delivered before the Medical Society on May 18th, by W. H. Allchin, M.D., F.R.C.P., Physician to the Westminster Hospital.

Among the many and varied phenomena presented to our observation by living beings, there is no one that recurs with such persistent frequency, no one that our experience leads us so inevitably to anticipate, as that such a being will in time, whether long or short, cease to exhibit those manifestations in virtue of which we speak of it as living, or, in other words, will die. It has seemed to me that I might, and I hope without too great presumption, profitably occupy the occasion which the courtesy of the council of this society has extended to me in putting before you some considerations which are connected with the investigation of this problem. For a thing to be dead implies, properly speaking. that the thing has lived, and the term is inapplicable, or should be were we precise in our language, to material that has not been just previously living. It will be impossible, therefore, to enter on the consideration of death without some preliminary agreement in notion of what is meant by life, which, after all, is only another aspect of the same problem.

We find that the essential facts of vitality as ascertained by observation, experiment, or experience are: (1) The invariable presence of a certain material of very easily recognised physical properties, but of a highly complex but as yet undetermined chemical composition and molecular structure known as protoplasm, which recent investigation has tended to show is not so homogeneous as was originally thought, though the details of its arrangement the microscope as yet has imperfectly revealed. (2) Such material occurs in isolated and distinct particles of exceeding minuteness, probably always, even in its simplest form, differentiated into cytoplasm and nucleus—the cell; or as aggregations of such particles, frequently in association with other materials less complex in composition and not possessing the properties we call life. (3) Given this substance, we call it living when it exhibits certain special forms of energy to which the collective term "irritability" may be applied, or more specifically "contractility," "nerve power," and "secretion." They are accompanied invariably by the liberation of heat and probably always, certainly often, by electrical manifestations, both of which are identical in nature and measurable by the same means as the heat of our fires or the electricity produced by our batteries. I am not concerned now to discuss how far these living energies are mutually convertible, or whether they are or are not correlated to the associated heat and electricity; it is sufficient to say that matter which presents these characters is called living, and unless they be present life is absent. For the display of these properties, and essentially included in the conception of life, is the presence in the cell of an adequate supply of non-living material—food, whereby its nutrition, as we say, is maintained. Regarded more closely, the three-fold functions above enumerated are the outcome of, or the expression of, protoplasmic nutrition, and as they are the essentials of vitality, nutrition and life may be to that extent regarded as synonymous terms.

But at the beginning and end of the subject, so to speak, are questions to which as yet but the most uncertain answer can be given. Given the living protoplasm, given this material with its vitality, how has it come into being, how does it come to an end? With the "why" as an explanation true science has no concern, but the "how" is its legitimate scope of inquiry.

Before proceeding to consider the nature of death, what is implied by it, and how it comes about, it is worth while to inquire whether death is an invariable termination to life, whether, in short, every being that lives does sooner or later die. If we confine ourselves to the higher forms of life, the reply would undoubtedly be "Yes," but this cannot be so readily affirmed if we regard the simplest forms of existence. Here we note a close connection between the mode of origin of life and the conditions of its termination. Universal experience shows that such beings as propagate sexually, whether animals or plants, or, in other words, beings which originate from the union of distinct sexual elements and of themselves consist of aggregations of cells and cell derivatives resulting from this union, will sooner or later die, even though, so far as can be recognised, their surroundings and conditions of existence remain the same as when their vitality was at its zenith. On the other hand, those organisms which reproduce by a process of fission cannot, with a possible partial exception to be presently noted, be said to die, but continue their existence in their progeny, which primarily consists of the constituent material of their parents, the whole of the parent body being used up in the formation of the descendants. If we regard, as I think we are entitled to do, fissuring of a protoplasmic unit (cell) as the exhibition of an extreme and peculiar form of the power of contractility which is possessed in some degree by all matter that is living (induced by some stimulus the nature of which is quite unknown), but only manifested on certain occasions, then we bring the reproductive act of these sexual organisms into line with the common properties of living matter and facilitate its consideration and description. If each resulting portion of a fissured cell continues to exhibit after its separate existence the characteristics of all living material as possessed by its parent, its power of self-nutrition with all therein implied, and its powers of adaptability within limits to varying conditions, and in due time to subdivide into two portions, each again continuing the *rôle*, then indeed such beings cannot be said to die except by accident, and are essentially immortal. No greater difference in identity exists between parent cell and progeny than exists in the parent cell itself at successive periods of its own existence, consisting as that existence does of ceaseless changing with continuity of character. All unicellular organisms, however, do not appear to be capable of unlimited propagation by fission. It has been long known that the infusoria which are among the highest developed of the protozoa, do after a series of such multiplications gradually exhibit a slackness in the fissuring until at length the act is no longer repeated and the individual dies. But should the infusorian meet with another of its kind in a like state of enfeeblement, and a temporary conjugation between the two occur, they will then separate, reinvigorated with a new lease of vitality and continue their propagation by fission until again exhausted. Such a condition is one of less complete immortality than obtains in other protozoa and protophyta, but it falls considerably short of the inherent mortality that pertains to all forms of multicellular life. Among the metazoa and metaphyta by no means can senescence and death be averted, however favourable may be the conditions for their continued existence. But even then some multicellular organisms do not wholly die. The body of the individual undoubtedly perishes, but the sexual elements contained therein, and for the production and care of which the individual soma finds its chief *raison d'être*, whereby the species may be maintained, continues to live, given only suitable conditions, and the fertilised ovum resulting from these elements, consisting as it does of integral parts of the parents, develops and grows, thus maintaining from generation to generation structural continuity. Death, therefore, which is a condition only known in the higher divisions of living beings, comes to mean a cessation of some only of the activities possessed by these beings, the greater proportion of their activities it must be admitted, and those which concern the maintenance and wellbeing of the individual as opposed to those by which the race is continued. It is the mass of the body that dies; that which lives, or can live, conditions being favourable, constituting but a minute fragment compared thereto, in the highest forms of all, a mere particle, and this participates in its partner's death unless removed to suitable surroundings. I need scarcely say that I make no reference to organisms being killed, that is to say, being subjected to unfavourable conditions of the environment. Countless myriads of immortal protozoa die, but that is due to the nature of their surroundings, they do not contain within themselves the inevitable destiny of death which they cannot avoid. Similarly myriads of the equally immortal sperm and germ elements of the higher and highest developed forms perish from like causes. My remarks obviously apply to death as a natural phenomenon, and as such it has a somewhat more restricted scope than at first appears.

We are, then, in the position to make these general statements: 1. That death as an incident in the evolutionary cycle is not inevitable to all living beings. 2. That whilst unicellular organisms are immortal those of any higher grade of structure of inherent necessity die. 3. That these latter are, given favourable conditions, continuously propagated by specialised portions of their own substance, the individual itself, apart from these portions, perishing. 4. That the power of self-division and hence of perpetuation with avoidance of death is lost by cells which have advanced beyond the most rudimentary stages of differentiation, such power being restricted to those elements which retain their embryonic character and most completely by those which form the sexual elements.

From such conclusions, then, naturally follows the question which is at the root of the subject, how is it that the vital processes decline and cease after a manifestation over a tolerably definite period, more or less peculiar to each species of animal and plant, peculiar to them in the same sense as a specific size or shape is peculiar? What is the immediate antecedent to the cessation of vital activity as invariably seen in sexually propagating organisms? Whence comes, and what is the nature of the breaking strain which disrupts the ceaseless interchanges which constitute life? It may be at once admitted that no satisfactory answer can be given.

Conceiving as we do that the energies of vitality are the sensible expression of continuous interchange in a highly complicated material, one phase of which is a building up, or assimilation, to be succeeded at some higher degree of complexity and instability by a breaking down and dissimilation, is it in the anabolic stages that the inherent antecedent that finally arrests the metabolism, and with it life, is to be found? or is it that in the catabolic descent that materials may be formed which exert a slowly increasing pernicious influence on the entire process, autogenetic poisons, in fact, that are invariable accompaniments of protoplasmic change? or, lastly, may it be some failure in the stimulus, the existence of which is involved in our conception of this same protoplasmic activity? Conformably with our fundamental hypothesis of the nature of nutrition it is difficult to see any other direction in which to look for our desired antecedent. Expressed in terms more in accordance with our grosser conceptions of physiology, does death result from some imperfection in the nutritive material (ingesta) or in some toxic product of waste (egesta), or for want of the needful stimulus to the very changes which evolve the material and determine the products? That it is the soma that dies and that the life of the soma or individual is identical with its nutrition would, consistently with our present hypothesis, make it probable that somewhere within these concepts what we are seeking is to be found.

In regard to the question of stimulus, there is one circumstance which seems to bear a very constant relation to the onset of death, viz., the reproductive effort. In many beings this effort is made but once, and then, as a rule, death soon follows. In others the effort is capable of being repeated over a period (maturity) of varying length, but when it fails senescence sets in. A review of all the circumstances would seem to show that failing vitality follows the cessation of reproductive capability rather than that this latter fails because of approaching death, and, if we may speculate with others, it appears to be among the possibilities that the lacking stimulus is to be found in connection with the sexual elements themselves—some ferment, may be, such as we assume to exist in those lately recognised internal secretions, the potency and influence of which in respect to nutritive processes we are but just gaining a glimpse of. An analogue for my suggestion is, perhaps, to be found in the case of the thyroid and thymus glands which exercise a profound control over tissue nutrition and even over growth itself. So may it be with the reproductive organs and their products. Exhausted by their efforts to perpetuate the individual, they no longer liberate that stimulus (call it provisionally ferment, internal secretion, or what you will till more precise knowledge defines it) which is needful for the maintenance of the nutritive processes of maturity, and for lack of which the individual deteriorates and dies. May it be that there is a real truth embodied in the experiments of Brown-Séquard, however incomplete they have hitherto been in results?

Based on some such considerations as these I have put forward must be, I take it, the full comprehension of the nature of death as we are in the habit of witnessing it at the bedside. We speak of cardiac failure, of fatal intoxication, whether from within or without, of arrested nerve influence, and the like, but the real meaning of such phrases, imperfect as they are, and often inadequate to explain why death should have happened, must be somewhere in the region I have now been groping in. Moreover, the very foundations of prognosis, if not of treatment, so far as this may attempt to modify nutritive processes by drugs or other reagents, must be built upon an understanding of the intimate nature of life and the inherent causes of its failure and cessation.

